# Virtual Reality as a Vehicle to Empower Motor-Cognitive Neurorehabilitation

**DOI:** 10.3389/fpsyg.2018.02120

**Published:** 2018-11-02

**Authors:** Daniel Perez-Marcos, Mélanie Bieler-Aeschlimann, Andrea Serino

**Affiliations:** ^1^MindMaze SA, Lausanne, Switzerland; ^2^Leenaards Memory Centre, University Hospital of Lausanne, Lausanne, Switzerland; ^3^Department of Clinical Neurosciences, University Hospital of Lausanne, Lausanne, Switzerland; ^4^Center for Neuroprosthetics, Ecole Polytechnique Fédérale de Lausanne, Geneva, Switzerland

**Keywords:** virtual reality, neurorehabilitation, motor-cognitive training, motivation, empowerment, neuroscience, stroke

## Abstract

In this paper, we advocate the combination of four key ingredients that we believe are necessary to design long-lasting effective treatments for neurorehabilitation: (i) motor-cognitive training, (ii) evidence-based neuroscience principles, in particular those related to body perception, (iii) motivational games, and (iv) empowerment techniques. Then, we propose virtual reality (VR) as the appropriate medium to encompass all the requirements mentioned above. VR is arguably one of the most suitable technologies for neurorehabilitation able to integrate evidence-based neurorehabilitation techniques and neuroscience principles into motivating training approaches that promote self-management by empowering patients to own their recovery process. We discuss the advantages and challenges of such an approach on several exemplary applications and outline directions for future developments. We strongly believe that the combination of positive psychology and positive technology mediated by VR-based interventions can heavily impact the rehabilitation outcomes of motor-cognitive functions along all the stages of the rehabilitation path.

## Introduction

Stroke remains a leading cause of long-term disability worldwide, making the improvement of post stroke outcomes a major healthcare objective. According to the American Heart Association (AHA), in 2014 the prevalence of stroke in the United States was 2.8%, with a projected increase of 20–30% by 2030 (Benjamin et al., 2017).

Among the multidimensional impairments of stroke (physical, cognitive, affective, and social), paralysis has historically centered major research investment. However, there is a close relationship between motor and cognitive deficits post stroke. For instance, spatial neglect severity, as observed in the activities of daily living, is a significant and independent predictor of upper limb outcome in right hemispheric stroke patients (Vanbellingen et al., 2017). In a prospective study contrasting functional independence and cognitive assessments at two time-points, Jokinen et al. (2015) showed that, even with a good recovery 3 months post-stroke (modified Rankin Scale = 0–1), 69% of first-ever-stroke patients suffered from cognitive impairment affecting one (25%), two (15%) or multiple (32%) domains. In addition, the presence of cognitive impairments was associated with functional dependence 15 months post-stroke. In another longitudinal study, depression and cognitive impairment at 3 months were significantly associated with disability 5 years post stroke (Yang et al., 2016).

Recovery is a long-term process that usually lasts longer than rehabilitation services (Demain et al., 2006). Longitudinal studies have reported that only 34% of stroke survivors are functionally independent 5 years after the accident (Wilkinson et al., 1997). Hence, the important of providing effective home-based solutions that focus on both clinical but also adherence goals. Indeed, evidence shows that the success of any therapeutic intervention is directly related to patient motivation (Maclean et al., 2000). Importantly, the level of adherence to home-based physical training programs is directly correlated to the level of physical activity before the treatment, as well as to the adherence to the training sessions in the clinic (Jack et al., 2010). In particular for stroke, evidence shows that adherence to home-based exercise programs is low, with lack of motivation consistently appearing among the main causes. Other reported barriers include musculoskeletal issues, fatigue (Jurkiewicz et al., 2011), mood disorder (Kim, 2016) and “dependence of therapist” (Ogwumike et al., 2014).

Neurorehabilitation programs inspired from positive psychology, which focuses on the bio-psycho-social aspects of cognitions, emotions, and positive experiences (Riva et al., 2012), may reveal particularly compelling. They can help promote self-confident, self-management and, thus, independence. When integrated into effective training programs, they can boost the rehabilitation outcome and significantly increase the quality of life of stroke survivors (Fryer et al., 2016).

In this position paper, we advocate the combination of four key ingredients that we believe are necessary to design long-lasting effective treatments for neurorehabilitation: (i) motor-cognitive training, (ii) evidence-based neuroscience principles, in particular those related to body perception, (iii) motivational games, and (iv) empowerment techniques. Then, we propose virtual reality (VR) as the appropriate medium to encompass all the requirements mentioned above. Finally, we discuss the advantages of such an approach on several exemplary applications and outline directions for future developments. For the sake of clarity, this paper is not a review of the available evidence on the efficacy of VR in neurorehabilitation – few review and meta-analyses papers are available (Lohse et al., 2014; Corbetta et al., 2015; Dockx et al., 2016; Gibbons et al., 2016; Massetti et al., 2016; Laver et al., 2017). The cited papers are functional to discuss the aforementioned aspects.

## The Synergetic Effect of Adaptive Motor-Cognitive Training

Up to 75% of stroke survivors suffer from upper limb movement disabilities (Henderson et al., 2007). In addition, up to 80% suffer from cognitive deficits affecting attention, perception or behavior control (Gottesman and Hillis, 2010), which persist 6 months after the vascular event in up to 70% of cases (Mellon et al., 2015). On the other hand, motor rehabilitation cannot proficiently be performed by patients with specific cognitive deficits (Dennis et al., 2011). Finally, such cognitive deficits are strong negative predictors not only of functional recovery (Robertson et al., 1997) but also of quality of life (Barker-Collo et al., 2010). Therefore, training programs should target both cognition and motor recovery, if possible addressing problems encountered in activities of daily living. For instance, one could train reaching (and other related) movements with the affected arm for cooking a sandwich in combination with the problem solving associated with the task (e.g., sequencing, planning).

It is important to tailor any intervention to both cognitive and motor levels of impairment. A preliminary assessment should clearly delineate the profile of motor and cognitive deficits. There is increasing evidence that physical exercise also contributes to improving cognitive functions post stroke (Cumming et al., 2012; Oberlin et al., 2017). Training both motor and cognitive functions simultaneously (instead of in separate sessions) would also increase therapy efficiency, maximizing the amount of therapy (dose) delivered for both modalities while reducing risk of fatigue. The benefits of combining physical and cognitive exercise go beyond recovery itself, as it may enhance overall well-being and reduce multiple risk factors responsible for recurrent strokes and coronary events (Tiozzo et al., 2015). Combination of motor and cognitive training has been shown to also reduce length of hospital stay (Kalra et al., 1997).

## Integration of Evidence-Based Neuroscientific Principles

The 70% recovery rule has disrupted the neurorehabilitation field and has put the focus on the optimization level of current neurorehabilitation procedures and practice (Prabhakaran et al., 2008; Krakauer and Marshall, 2015). The question arises, however, whether we can do better (beyond that 70%), especially if we can predict recovery chances of stroke survivors. To this aim, neurorehabilitation programs ought to combine evidence-based clinical neurorehabilitation strategies and neurosciences principles. Within that context, few neuroscientific principles have been proposed for motor rehabilitation. Here, we highlight those targeting multisensory stimulation, the mirror neuron system and motivation.

Decades of research in fundamental neurosciences have shown that our senses perform better in the activities of daily living when they are provided with multisensory stimuli (Johansson, 2012). Congruent multisensory stimulation will reinforce reflexes and automatisms whereas incongruent inputs will slow down the responses while requiring higher attentional resources. However, most popular technological solutions for cognitive neurorehabilitation are based on videogame-like (or serious game) approaches, where patients move in front of a console and only receive visual feedback about their movements (often via abstract content not related to the own body). This represents a limited approach, whereby the feedback to the user is limited to a single sensorimotor action-perception loop: the patient moves and receives only visual feedback from the screen.

Since stroke survivors often present sensory loss, it is necessary to propose congruent, multisensory environments that stimulate not only visuomotor coordination but also the somatosensory system, e.g., via tactile and proprioceptive training (Aman et al., 2015). Likewise, multisensory stimulation can include congruent audiovisual stimulation. For instance, in a two-arm study with patients with hemianopia (a defective vision or blindness in half of the visual field), the group following audiovisual training with spatially matching sounds improved visual detection and exploration, oculomotor scanning and activities of daily life, whereas the visual training group did not (Passamonti et al., 2009).

In stroke rehabilitation, priming (a type of implicit learning) of the motor cortex is associated with changes in neuroplasticity that are associated with improvements in motor performance (Stoykov and Madhavan, 2015). Mirror neurons found in key motor areas respond not only during action execution but also to action observation, i.e., the motor system may be activated without overt movement, which enables training interventions also in case of severe hemiparesis (Garrison et al., 2010; Buccino, 2014). Together with related techniques like motor imagery and mirror therapy, these approaches have proven effective as potential adjuncts to motor training -with the caution that many articles include small samples and there may be a publication bias as adequate control conditions are sometimes missing (Deconinck et al., 2015)-, even though individual factors that may predict response to treatment (e.g., lesion size, co-morbidities, psychosocial) are still to be identified (Stoykov and Madhavan, 2015).

Neurorehabilitation interventions can also benefit from neuroplasticity enhanced by rewards. In particular, the ventral striatum has been suggested to act as a common motivational node able to switch connectivity between motor and cognitive circuits depending on the task demand: the same extrinsic reward (coin images) can trigger the recruitment of cognitive (by increasing caudate nucleus activity when the task is mentally demanding) and motor regions (by increasing the putamen activity when the task is physically demanding) (Schmidt et al., 2012). Rewarding tasks that combine both motor and cognitive demands could therefore lead to activation of both motor and cognitive pathways. Altogether, basing neurorehabilitation programs on current neuroscientific understanding and transferring them into clinical practice via personalized treatments (e.g., enabled by technology-mediated interventions), could therefore help boost recovery.

## Enjoyment and Motivation Through Gamification

Determining the training features to the patient’s pace of recovery not only maximizes training potential, but also prevents habituation and frustration. This facilitates keeping patient motivation at an optimal level during the long rehabilitation process. Neurorehabilitation programs should, thus, inspire from Seligman’s “PERMA” theory (i.e., positive emotions, engagement, relationships, meaning and achievement; Seligman, 2011) and include activities that enable the patient to float in their Flow Zone, defined as where the person is at a high level of enjoyment with a balance between the difficulty of the task and the abilities of the person (Csikszentmihalyi, 1990). In this state of flow, the person feels comfortably challenged and highly engaged by the task feeling high levels of enjoyment. Maintaining a state of flow is important for promoting patient adherence to treatment, especially for home-based rehabilitation.

Repetitive routines of same actions of current rehabilitation programs (either motor or cognitive) do not contribute to the endurance of patient motivation in the long term. The impact of physical activity can be strengthened when training activities go hand in hand with enjoyment (Hagberg et al., 2009). Enjoyment through computer (serious) games is likely the most common approach for healthcare applications, ranging from behavior change (Baranowski et al., 2008) to rehabilitation (Nap and Diaz-Orueta, 2013).

For instance, Anguera et al. (2013) showed that, by using adaptive computer games to provide fully controlled dual-task training based on principles of cognitive control, it was possible to improve multitasking abilities in neurologically healthy elderly individuals, with gains persisting for 6 months. Personalized gamified tasks can also help strengthen brain modulation through variable attentional demands. Indeed, strategic divided attentional training programs with variable task foci have been suggested to recruit larger brain networks than single repeated practice. This could help prevent cognitive decline in healthy older adults and for those involved in rehabilitation of individuals with brain damage (Belleville et al., 2014).

Appropriate and timely feedback (e.g., encouraging messages) together with online adaptation of difficulty levels (matching patients’ needs and capabilities at every moment) can boost patient motivation. This can be achieved by setting realistic objectives, not too easy and not too hard, to maintain patients’ motivation as long as possible. Another technique is to introduce both unexpected events (that generate reactions of surprise that capture attention), mechanisms of control (if patients have the impression they control the situation, then they will remain proactive), and rewards (both online and offline). Rewards need to vary as well to reinforce vigilance and an interest in understanding *why* and *when* they are given. Frank et al. (2004) have shown that rewards should be tailored to the patient pathology and to the prescribed treatment. For example, Parkinson patients off medication would be more sensitive to negative rewards than those undergoing dopamine treatment. Thus, modulating positive and negative feedback accordingly should lead to optimal reinforcement learning processes. In sum, a clever interplay between therapeutic goals and gamification tools should be taken into consideration to maximize outcomes (Mader et al., 2016).

## Patient Empowerment

As their recovery progresses, it is important that patient autonomy is encouraged in order to reduce patient (and caregiver) dependence, especially when the return to home is approaching. Once back home, patients usually have to exercise on their own or with a fading supervision provided by both the caregiver and the therapist, with the latter sometimes remotely connected. Thus, training strategies should encourage patients to progressively manage their own motor and cognitive recovery as a motivational drive. This would have cascading effects on therapy intensity, duration and adherence, resulting in long-lasting positive outcomes. These, in turn, may transfer to real life and contribute to attaining a level of autonomy required for living independently.

“Patient empowerment is a core principle of patient-centered care and reflects one’s ability to positively affect his or her own health behavior and health status” (Govender et al., 2015). It is thus crucial to teach a patient to admit that they can influence their own health. This should be done by first giving them the knowledge of *what* impairments they are suffering from, *when* they will encounter difficulties in their daily living activities and *how* they can deal with them by using different strategies (educational content). This process aiming at self-management involves both meta-cognitive (i.e., having both knowledge and consciousness of the problem) and learning strategies to face the daily living difficulties resulting from cognitive and motor deficits. Recent meta-analyses and systematic reviews on self-management programs for stroke survivors living in the community have shown reliable evidence that such programs increase participation, functional ability, and have positive effects on their quality of life (Warner et al., 2015; Fryer et al., 2016). Hence, these benefits are best transferred to daily life only when the program is personalized (mode of delivery, frequency, duration). In an empowerment intervention on stroke patients’ self-efficacy, Sit et al. (2016) found that empowered patients reported better functional recovery, especially in activities of daily living, and better self-management of their cognitive symptoms than a control group receiving standard therapy.

## Virtual Reality as a Vehicle to Boost Motor-Cognitive Neurorehabilitation

VR is likely today’s most powerful experiential technology available, i.e., a technology able to create immersive live experiences. One of the most recurrent terms associated with VR is “videogame,” particularly in the entertainment industry. Besides positively affecting motivation and enjoyment of training, videogames also impact cognition. In particular, playing action videogames (i.e., games that emphasize physical challenges, e.g., hand–eye coordination and reaction time) has been shown to robustly enhance attention and spatial cognition (Bediou et al., 2018). Exergames (i.e., physically active videogames) have been shown to improve cognition in both clinical and non-clinical populations, including executive functions, attention and visuospatial skills (Stanmore et al., 2017). VR can boost these effects by delivering those games in highly immersive and interactive environments and, importantly, tapping mechanisms of intrinsic (i.e., autonomous) and extrinsic (e.g., arising from rewards) motivation (Howard, 2017). For instance, one could imagine an immersive VR cooperative or competition task for promoting intrinsic motivation while reinforcing extrinsic motivation through gamification elements like progression scores and customization of the environment or virtual character. Those patients proud of their performance and progress and may be willing to share it with their close family and friends to reinforce their interindividual relationship.

From a neuroscientific perspective, VR offers multisensory stimulation able to evoke the mirror neuron system and mechanisms of action observation, among other therapeutic techniques, which have been suggested to be effective for motor recovery (Stoykov and Madhavan, 2015). VR represents a unique medium for neurorehabilitation where participants can embody a virtual body that can gather synthetic multisensory (visual, motor, touch) stimulation (Perez-Marcos et al., 2012). This stimulation versatility can be used to systematically modulate (stimulate or attenuate) activity in specific brain regions (You et al., 2005; Adamovich et al., 2009). Virtual embodiment (i.e., the illusion of having and feeling as if real a virtual body in VR) is a critical factor in VR experiences that can be exploited to improve not only visuomotor coordination (as shown in motor rehabilitation post stroke), but also for cognitive-related deficits such as spatial attention impairments, executive dysfunction or memory loss. For instance, by adding body manipulations through embodiment illusions in an immersive environment, the eventual affected spatial perception of a stroke patient could be challenged, e.g., by means of a visuomotor adaptation task.

Regarding multisensory stimulation, interactive technologies like VR are best placed to incorporate closed-loop mechanics of challenge adaptivity with gaming environments and enriched multisensory feedback (Mishra et al., 2016). Such enriched virtual environments have the potential to optimize motor learning by manipulating practice conditions that explicitly engage motivational, cognitive, motor control, and sensory feedback-based learning mechanisms (Levin et al., 2015). For example, people with Parkinson disease showed similar improvement in reaching performance after training to respond to moving balls both in VR and physical environments by using appropriate cueing speed (Wang et al., 2011). Real-time movement feedback can also be used to reinforce control of movement parameters, e.g., joint trajectory, and to reduce compensation movements, e.g., excessive trunk displacement (Subramanian et al., 2013). Adding vibrotactile feedback on tubes simulating oars in a VR-based rowing game, with an avatar representing the user’s arms rowing from a first-person perspective, can help convey the illusion of movement to the user (Vourvopoulos et al., 2016). Vibrotactile feedback can also be used to improve the motion symmetry on stroke rehabilitation (Hung et al., 2015). Coincident audiovisual spatial feedback provided in a head-mounted display environment could help patients with hemispatial neglect to regain a coherent sense of space. This feedback can extend to other behavioral, cognitive, and emotional changes captured by dedicated sensors. Making these inputs explicit for patients gives them the opportunity to become aware of their problem (meta-cognition) and progress, which will favor patient autonomy in the long term and, thus, their empowerment.

VR-mediated motor-cognitive interventions can help retain and transfer to real life the outcomes obtained during the training period. For example, a longitudinal study with community-living older adults compared the effectiveness of motor versus motor-cognitive training, the motor component mediated by a treadmill and the cognitive component by non-immersive VR, to reduce the number of falls. The virtual environment imposed a cognitive load that demanded attention, planning and dual tasking. The effect was higher in the motor-cognitive group, with incident rate of falls continuously decreasing to half at 6 months after completion of the training (Mirelman et al., 2016). Personalization of the training content into the scope of patients’ daily living activities can contribute to the transfer of training outcomes and to promote autonomy in activities of daily living, e.g., by training cooking activities in a virtual kitchen (Foloppe et al., 2018) or shopping in a virtual supermarket (Rand et al., 2009).

A critical feature for an effective rehabilitation program is the possibility of adapting the training based on the patient’s needs and performance. When applied to motor rehabilitation post stroke, VR easily allows progressively adapting the training program via automated systems based on the patient’s performance, for adapting the task difficulty levels (Ballester et al., 2016), or based on reinforcement-based therapies, for counteracting learned non-use of the impaired limbs (Ballester et al., 2015). In addition, and to lead to further improvements in performance (Anguera et al., 2013; Shin et al., 2016), adapting the motor and cognitive load can also help face different rehabilitation challenges, such as fatigue and adherence. For instance, a VR-based training program could progressively challenge patients with hemispatial neglect by adapting the difficulty of a visual scanning task and lateralizing the reward amount to drive patient attention to the neglected hemispace.

Kinematics and other objective measures provided by VR systems (e.g., motion capture, eye tracking) can be used for adapting the difficulty level of the tasks. Moreover, VR technology can also integrate physiological signals allowing monitoring the level of arousal or affective state (e.g., via galvanic skin response, heart rate, EEG). This information can be used to adapt the training, in real time, to the patient’s status (at all three levels: physical, cognitive, and affective) in order to perpetuate the patient’s feeling of completeness and energized engagement in the therapeutic activity. Ultimately, the training program should offer patients the opportunity to select the virtual environment/task that motivates them or better fits their interests and values, as well as its personalization (e.g., favorite color, avatar appearance). For instance, for bed bound patients, immersion into outdoor environments of their choice could represent a way to virtually leave the hospital for a while. Further approaches should also consider the integration of technologies able to read people’s emotions, e.g., based on facial expressions or other neurophysiological markers, to enhance motivation levels.

Finally, VR-mediated rehabilitation programs provide an ideal means to ensure the continuum of care, from early stages at hospital to home-based interventions and follow-up. VR is destined to become ubiquitous in our society thanks to the mass-market arrival of ease-of-use and affordable devices, which are perfectly eligible for home treatment. VR platforms are easily scalable: measurement (motion capture, EEG, instrumented objects, etc.) and stimulation (head-mounted display, haptic feedback, functional electrical stimulation, etc.) instruments as well as accessories required at each rehabilitation stage and by each individual intervention can be integrated as required. In particular, any data collected can be remotely accessible to clinicians, therapists and caregivers to monitor patient performance and behavior, to act consequently (e.g., updating the training program, providing assistance).

### How to Exploit Performance Measures for Objective Motor and Cognitive Assessment

The same neurophysiological and behavioral information collected for adapting difficulty levels and other parameters in VR-based training can also be applied for assessment of the treated deficits. For instance, head gaze and motor performance during exposition to an ecological task for evaluation of the far space in a virtual environment can extend the evaluation of stroke patients with unilateral spatial neglect. Preliminary results suggest that ecological immersive VR-based assessments could help detect unilateral spatial neglect in chronic patients who do not show signs of neglect in standard paper-and-pencil assessments (Ronchi et al., 2018).

Extending the use of VR to assessments presents at least three main advantages: (i) technologies inherently used in VR-mediated solutions can provide additional and objective measures to expand today’s clinical assessments, e.g., using motion capture technology to evaluate range of motion based on acquired kinematic information of the limb movements (Shishov et al., 2017); (ii) integration of these assessments into the training programs can help develop “smart” protocols that optimize recovery and learning, e.g., determining specific attentional deficits in a real-world simulated scenario (something not possible with paper-and-pencil assessments) in order to prescribe the right training; and (iii) as VR-mediated medical devices will populate hospitals and clinics and, consequently, databases of clinical data will grow, new assessment standards may be created. Their cross-validation with clinical and other neurophysiological markers (e.g., neuroimaging) may help identify advanced predictors of recovery that may be used to personalize treatments, e.g., by means of machine learning algorithms.

### Challenges of VR-Based Systems for Neurorehabilitation

All the above-mentioned potential advantages can be put best into practice with optimal VR systems. Nowadays, however, VR technology is not equally developed in all its domains. For example, while visual displays are highly immersive (with high resolution and low latency) and affordable (in a minimalistic form, just a cardboard and our smartphone are necessary), other sensory inputs and actuators are not mature yet. Regarding tactile haptic rehabilitation, there is still a need for both scientific and technological developments to ensure its full potential is realized (Demain et al., 2006). Similarly, integration of olfactory and taste senses is still in its infancy (Spence et al., 2017).

The second big challenge is building clinical credibility. Despite the existence of several reviews and meta-analyses on VR for stroke (Lohse et al., 2014; Gibbons et al., 2016; Laver et al., 2017), Parkinson disease (Dockx et al., 2016), multiple sclerosis (Massetti et al., 2016) and multiple neurological conditions (Cano Porras et al., 2018), results about the effectiveness of VR-mediated training are yet inconclusive. One main reason for this is that many studies include small sample sizes as compared to the great variability of the tested clinical populations, which makes extremely difficult to control for other variables such lesion location and size, severity of impairments, phase of recovery and, importantly for cognitive functions, no separation between age or level of education (Fetta et al., 2017). Another key factor is the choice (or lack) of the control group to compare the effect with: for instance, for home rehabilitation, if the effectiveness of a VR-mediated training (with the therapist remotely shaping and monitoring the training program) is compared to that of 1-to-1 therapy sessions of the same intensity and duration with a highly trained clinician, the non-inferiority of the VR-mediated training should be actually considered as a positive outcome, independently of other socio-economic advantages such as reduced costs. Conversely, in motor rehabilitation for stroke patients, VR has been introduced with the aim of allowing increased rehabilitation dosage compared to traditional therapies. In this vein, there is encouraging evidence that VR may improve upper limb function in hemiplegic patients when added to standard physical therapy (Laver et al., 2017). However, only few studies have focused on the use of VR-mediated interventions for cognitive neurorehabilitation. Indeed, people with significant cognitive impairment are excluded from most studies of VR-mediated motor rehabilitation, making unable to pool results for cognitive function and, thereby, preventing comprehensive conclusions about how applicable VR-mediated interventions are to a wide range of stroke survivors (Laver et al., 2017). A recent feasibility study comparing both standard therapy and VR-mediated solutions has shown additional difficulties of subacute stroke patients to recover physically and cognitively when they present mild cognitive impairment, and particularly signs of depression (Cameirão et al., 2017). In a 1-month randomized controlled trial with 18 chronic stroke survivors, the VR group showed significant improvements in global cognitive functioning, attention, memory, visuospatial abilities, executive functions, emotion and overall recovery: a between groups analysis showed significantly greater improvements in global cognitive functioning, attention and executive functions when comparing VR-mediated to conventional therapy (Faria et al., 2016). However, that study did not include follow-up or measure of transfer to real life. Therefore, although recent studies start taking some of these aspects into consideration (e.g., Faria et al., 2018), additional larger and high-quality studies with stratification including different conditions are needed. Those studies should target the role of the diverse mechanisms underlying the therapy (e.g., the visual feedback, the multisensory integration, the embodiment), rather than comparing just VR versus other therapies. This would help us understand what is(are) the effective element(s) for each patient profile (based, for instance, on lesion size and location, deficit severity), how and under which conditions (how, how much, when, how often) they should be delivered.

## Conclusion

In summary, VR is arguably one of the most suitable technologies for neurorehabilitation, able to integrate evidence-based neurorehabilitation techniques and neuroscience principles into motivating training approaches that promote self-management by empowering patients to own their recovery process. We strongly believe that the combination of positive psychology and positive technology (Riva et al., 2012) mediated by VR-based interventions can heavily impact the rehabilitation outcomes of motor-cognitive along all the stages of the rehabilitation path.

## Author Contributions

DP-M and MB-A drafted the manuscript with important contributions from AS. All authors participated in the review and revision of the manuscript and have approved the final manuscript to be published.

## Conflict of Interest Statement

DP-M, MB-A, and AS are employees of MindMaze SA.
